# Flower vinegar prepared from Yunnan large-leaved tea tree prevents high-fat diet-induced obesity in mice by regulating gut microbiota

**DOI:** 10.3389/fnut.2026.1749951

**Published:** 2026-03-18

**Authors:** Wenshu Peng, Wei Yang, Li Ma, Qiaomei Wang, Ruijuan Yang, Aibing Ji, Mingyue She, Tao Wang, Wanying Gong, Liang Yan

**Affiliations:** 1 Puer Institute of Pu-Erh Tea, Pu'er, China; 2 College of Pu'er Tea, West Yunnan University of Applied Sciences, Pu'er, China; 3 College of Tea Science, Yunnan Agricultural University, Kunming, China; 4 College of Food Science and Technology, Yunnan Agricultural University, Kunming, China

**Keywords:** gut microbiota modulation, high-fat diet, metabolic disorders, obesity prevention, tea flower vinegar, Yunnan large-leaved tea tree flower

## Abstract

Obesity and its metabolic complications are major public health concerns. The gut microbiota plays a pivotal role in regulating host adiposity. Fermented products from *Camellia sinensisvar*. *Assamica* (Yunnan large-leaved tea) flowers, a novel food ingredient, may offer therapeutic potential, but their effects on obesity and gut microbiota remain unexplored. We investigated the anti-obesity effects of vinegar fermented from *Camellia sinensisvar*. *Assamica* flowers (TTFV) in a high-fat diet (HFD)-induced obese mouse model. Body weight, glucose and lipid metabolism, hepatic injury, steatosis, inflammation, and oxidative stress were assessed. Metabolomic analysis and metagenomic sequencing of gut microbiota were performed. Key metabolic pathways were analyzed. TTFV supplementation significantly attenuated HFD-induced body weight gain, improved glucose and lipid profiles, alleviated hepatic steatosis and injury, and reduced systemic inflammation and oxidative stress. TTFV modulated host metabolite profiles and related metabolic pathways. Crucially, TTFV reshaped the gut microbiota structure: it increased the relative abundance of *Bacteroidota* and decreased the *Firmicutes/Bacteroidota* ratio at the phylum level. At the family level, it promoted beneficial bacteria (*Oscillospiraceae, Eubacteriaceae*) and suppressed potentially harmful ones (*Erysipelotrichaceae*). Metabolic pathway analysis indicated TTFV's positive role in maintaining cellular homeostasis and regulating metabolic disturbances. Our findings demonstrate that TTFV exerts protective effects against HFD-induced obesity in mice. These benefits are closely associated with the remodeling of gut microbiota composition and the modulation of key metabolic pathways. This study is the first to report the anti-obesity potential and microbiota-regulating effects of TTFV, suggesting its promise as a functional food ingredient for promoting intestinal health and mitigating obesity-related metabolic disorders.

## 1 Introduction

Obesity has become a major global public health challenge in the 21st century. According to the latest prediction of the World Obesity Federation, by 2030, more than 50% of adults will become overweight or obese (1). Current statistics indicate that the global number of overweight or obese individuals has exceeded 2 billion, with China ranking first worldwide in the number of obese people (2). Obesity is closely associated with metabolic syndrome, type 2 diabetes mellitus (T2DM), metabolic dysfunction-associated steatotic liver disease (MASLD), and cardiovascular disease. The pathological mechanisms of obesity involve multiple interacting factors, including chronic inflammation, oxidative stress, insulin resistance (IR), and gut microbiota dysbiosis (3). Consequently, exploring effective obesity intervention strategies to safeguard public health has become a current research focus.

In recent years, bioactive components of natural plants have become a research focus in obesity intervention due to their multi-target regulatory properties and low side effects. Tea tree flowers have attracted increasing attention because they are rich in bioactive substances such as tea polyphenols, tea polysaccharides, and flavonoids (4, 5). Previous research has shown that tea tree flowers contain significantly higher levels of polysaccharides than fresh tea leaves, and that can significantly improve the intestinal barrier function in immunosuppressed mice and inhibit inflammatory responses via the TLR4/MyD88/NF-κB pathway (6, 7). In addition, flavonoids of tea tree flowers reduce inflammatory infiltration of adipose tissue by scavenging reactive oxygen species (ROS) and inhibiting NF-κB activation (8). These findings suggest that bioactive components of tea tree flowers may ameliorate obesity-related metabolic abnormalities through synergistic actions of multiple pathways. Although the Yunnan large-leaved variety of *Camellia sinensis var. assamica* (*J. W. Mast*.) *Kitam*, a notable local resource, possesses significant antioxidant and metabolic regulatory functions; most research has concentrated on its leaves, leaving the development of its tea flowers largely unexplored (9).

As a traditional fermented food, vinegar is rich in various bioactive components, including organic acids, polyphenols, melanoidins, amino acids, and saccharides (10). Its composition and functional properties are collectively influenced by the raw materials, production techniques, and the complex chemical transformations, physical changes, and microbial metabolic activities that occur during the brewing process (9). Therefore, this study fermented tea flowers into tea flower vinegar, which is expected to retain the advantages of tea flower bioactive components while incorporating the characteristics of acetic acid fermentation, maybe playing an important role in lipid-lowering, anti-inflammatory, and gut microbiota regulation (11).

This study aims to systematically evaluate the effects of tea flower vinegar on regulating lipid metabolism, alleviating oxidative stress, improving gut microbiota structure, and modulating inflammatory pathways through animal experiments, thereby exploring the potential of this fermented product in ameliorating obesity-related metabolic abnormalities. The findings provide a theoretical basis for the high-value utilization of *Camellia sinensis var. assamica* (*J. W. Mast*.) Kitam resources and lays the foundation for developing safe and effective obesity-intervention products.

## 2 Materials and methods

### 2.1 Preparation of Yunnan large-leaf tea flower vinegar

Flowers of the Yunnan large-leaved tea tree were harvested at the Tea Tree Seed Farm in Pu‘er City, China. After sun-drying, the flowers were crushed into powder and stored in a dry state until use. The fermentation process of Yunnan tea tree flower vinegar (TTFV) was as follows: dried tea flower material pulverized (solid-to-liquid ratio 1.5:100) + sucrose (7.0%) → 48h filtration to remove residue → addition of white liquor (7% vol) + kombucha (0.05%) → fermentation at 30 °C for 13 days → centrifugal and filtration → sterile processing yields TTVF (original solution), stored at 4 °C. The components and composition of TTFV are shown in Table 1.

**Table 1:** Components and composition of Yunnan large-leaved tea tree flower vinegar.

**Chemical composition**	**Content(mg/L)**
Protein	0.0067
Tea polyphenols	0.07
Tea polysaccharides	2.22
Catechins	1.31
Soluble sugar	2.13
Total flavonoids	0.01
Theobromine	7.81
Theophylline	0.97
Caffeine	45.04
Total saponins	0.25
Vitamin A	0.0021
Vitamin C	0.02
Total acid	0.57
Non-volatile acid	0.06
Soluble salt solids	3.37

### 2.2 Experimental design and animals

Thirty six-week-old male C57BL/6J mice, purchased from Henan Scripps (Henan, China), were housed under SPF-grade conditions (12 h light/12 h dark cycle, 25 ± 1 °C, 55% humidity). Three mice were housed per cage across two cages. After a 2-week acclimatization period with *ad libitum* access to food and water, mice were randomly divided into 5 groups (*n* = 6 per group) and fed with the following diets for 20 week. The concentration gradient of TTFV was established based on the results of preliminary *in vitro* experiments, including analyses of its inhibitory activities against α-glucosidase and lipase, and was appropriately adjusted with reference to the methods reported by Li (12, 13), The detailed experimental procedures and corresponding results of the *in vitro* assays are provided in the Supplementary material (Sections 1.1, 1.2), Supplementary Results (Sections 2.1, 2.2), and Supplementary Tables S1, S2. Specifically, the high dose was undiluted TTFV, the medium dose was 50% TTFV + 50% purified water, and the low dose was 33% TTFV + 66% purified water, yielding a ratio of high: medium: low = 3:2:1.The groups were defined as follows: 1) Normal control group (NCD diet, D12450J, 10% fat energy): standard diet supplemented with an equal volume of distilled water via oral gavage at 8:00 a.m.daily; 2) High-Fat Diet Group (HFD, 60% energy from fat): high-fat diet supplemented with an equivalent volume of distilled water via oral gavage at 8:00 a.m. daily; 3) High-Fat Diet + Low-Dose TTFV Group (LD): high-fat diet supplemented with TTFV 6.6 mg/kg/d via oral gavage at 8:00 a.m. daily; 4) High-fat diet + moderate-dose TTFV group (MD): high-fat diet supplemented with TTFV 10 mg/kg/d via oral gavage at 8:00 a.m. daily; 5) High-fat diet + high-dose TTFV group (HD): high-fat diet supplemented with TTFV 20 mg/kg/d via oral gavage at 8:00 a.m. daily. Experimental mice had free access to food and water. Daily measurements were taken of food intake and body weight gain. Experimental feeds were purchased from Shanghai Hongbai Technology Co., Ltd. (D12492). The basal diet composition was based on the Composition and Nutritional Levels of Mouse Basal Diets (2025). All animals were randomly assigned using a computer-generated sequence. Experimental protocols were approved by the Institutional Animal Care and Use Committee (IACUC) of Wuhan Seville Biotechnology Co., Ltd. (License number: SYxK (Hubei) 2023-0101) and conducted in strict accordance with the guide for the care and use of laboratory animals.

### 2.3 Sample collections

After 20 weeks of dietary intervention, mice were anesthetized with ethyl ether after food was withheld for 12 h. At this time, blood samples were taken from the inferior vena cava in heparin-coated tubes. Blood was centrifuged at 3,000 rpm for 15 min at 4 °C, and the supernatant was collected for biochemical assays. After blood was collected, the liver was removed, rinsed with physiological saline solution, and immediately stored at −80 °C in a deep freezer. Part of each liver was fixed in 10% neutral formalin for Oil Red-hematoxylin, while another part was embedded in Optimal Cutting Temperature (OTC) for ematoxylin and eosin. Cecal contents were collected in sterile centrifuge tubes. All samples were immediately snap-frozen in liquid nitrogen and stored at −80 °C until analysis. For SOD, GSH-Px, and MDA analysis, we froze 50 mg of liver tissue in liquid nitrogen and stored it at −20 °C.

### 2.4 Oral glucose tolerance test (OGTT)

The OGTT was performed 48 h before the termination of the experimental schedule. Mice were fasted for 12 h before the experiment. After the fasting period, body weight was measured. Fasting blood glucose levels (0 min) were determined using a blood glucose meter and corresponding test strips (An Yi Type, Sannuo Biosensor Co., Ltd.) with blood collected from the mouse tail. Subsequently, mice were administered a sterilized glucose solution (Shanghai Shenzhixin Biotechnology Co., Ltd.) via oral gavage at a dose of 2 g/kg body weight (14). Blood samples were collected from the tail at 30, 60, 90, and 120 min post-gavage to measure blood glucose levels (15). Mice were maintained in a conscious, quiet state throughout the experiment under stable environmental temperature. Final blood glucose values at each time point were recorded to plot glucose-time curves. The OGTT results are expressed in area under the curve (AUC) in order to determine the degree of impairment of glucose tolerance (3).

### 2.5 Serum biochemical analysis

Fasting blood glucose levels were measured by the glucose oxidase method using a GOD-PAD kit (Shanghai Rongsheng Biotechnology Co., Ltd., China). Serum concentrations of total cholesterol (T-CHO), triacylglycerol (TG), low-density lipoprotein cholesterol (LDL-C), and high-density lipoprotein cholesterol (HDL-C) were measured. The activities of aspartate aminotransferase (AST) and alanine aminotransferase (ALT) were determined. Tumor necrosis factor-α (TNF-α), interleukin-6 (IL-6), interleukin-10 (IL-10), interleukin-1β (IL-1β), superoxide dismutase (SOD), glutathione peroxidase (GSH-PX), and malondialdehyde (MDA), were analyzed.

### 2.6 Histopathological examination of livers

Select the sampling site in fresh mouse liver tissue while minimizing mechanical damage. Samples should be collected within 1–3 min, with tissue blocks sized approximately 1 mm3. A petri dish pre-filled with electron microscopy fixative should be prepared before sampling. Excise a small tissue block and immediately transfer it into the petri dish; trim it into 1 mm3 pieces using a scalpel while immersed in the fixative. Transfer the trimmed tissue blocks to a microcentrifuge tube containing fresh electron microscopy fixative for continued fixation, then store and transport at 4 °C. Rinse three times with 0.1 M phosphate buffer (PB, pH 7.4), 15 min per rinse. Post-fix with 1% osmium tetroxide (prepared in 0.1 M PB, pH 7.4) at room temperature in the dark for 2 h. Rinse three times with 0.1 M PB (pH 7.4), 15 min per rinse. Dehydrate tissues sequentially through a graded ethanol series (30%, 50%, 70%, 80%, 95%, 100%, 100%) for 20 min per step, followed by two 15-min washes with 100% acetone. In filtrate at 37 °C as follows: 1:1 acetone:812 embedding agent for 2–4 h; 1:2 acetone:812 embedding agent overnight; Pure 812 embedding agent for 5–8 h. Pour pure 812 embedding agent into an embedding mold, insert the sample, and incubate at 37 °C overnight. Polymerize the embedding mold in an oven at 60 °C for 48 h, then remove the cured resin block for subsequent use. Section the resin block into 60–80 nm ultrathin slices using an ultramicrotome, and collect sections onto 150 m copper grids coated with Formvar film.

Stain copper grids with 2% uranyl acetate (saturated alcoholic solution) in the dark for 8 min. Wash three times with 70% alcohol, followed by three washes with ultrapure water. Stain with 2.6% lead citrate solution in a CO_2_-free environment for 8 min. Rinse three times with ultrapure water and gently blot with filter paper. Place grids in a grid box and air-dry overnight at room temperature.

Examine sections under a transmission electron microscope (Nikon Eclipse Ci-L, Japan) and analyze acquired images using Image-Pro Plus 6.0 software (Media Cybernetics, Inc., Rockville, MD, USA).

### 2.7 LC-MS analysis of differential metabolites

Fecal samples from six mice were randomly selected from each group and analyzed by Meggie Bio. For liquid chromatography-mass spectrometry (LC-MS) analysis, 50 mg of each fecal sample was used. A technique similar to but slightly modified from the method previously reported by Wang et al. (16) was adopted. The specific steps are as follows: transfer 100 μL of fecal sample to a centrifuge tube (EP tube). After adding 400 μL of extraction solution (acetonitrile: methanol = 1:1, containing isotopically labeled internal standard mixture), vortex for 30 seconds, ultrasonicate in an ice-water bath for 10 min, and incubate at −40 °C for 1 h to precipitate proteins. Subsequently, centrifuge at 12,000 rpm [RCF = 13,800 ( × g), *R* = 8.6 cm] for 15 min at 4 °C. Transfer the supernatant to a new glass vial for analysis. Quality control (QC) samples were prepared by mixing aliquots of all sample supernatants.

The ultra-high-performance liquid chromatography (UHPLC) system (Vanquish, Thermo Fisher Scientific) was set up with a UPLC BEH amide column (2.1 mm × 100 mm, 1.7 μm) and was connected to a Q Exactive HFX mass spectrometer (Orbitrap MS, Thermo). The mobile phase consisted of an aqueous solution of 25 mmol/L ammonium acetate and 25% ammonia water (pH = 9.75; A) and acetonitrile (B). The autosampler temperature was set at 4 °C, and the injection volume was 3 μL.

The source conditions for the QE HFX mass spectrometer with electrospray ionization (ESI) are set as follows: sheath gas flow rate at 30 Arb, auxiliary gas flow rate at 25 Arb, capillary temperature at 350 °C, full MS resolution at 60,000, MS/MS resolution at 7,500, collision energy at 10/30/60 in nitrogen collision energy (NCE) mode, and spray voltage at 3.6 kV (positive ions) or −3.2 kV (negative ions).

The raw data were converted to mzXML format using ProteoWizard and processed for peak detection, extraction, alignment, and integration using an internally developed program. Subsequently, the internal MS2 database (BiotreeDB) was applied for metabolite annotation. The cutoff value for annotation was set at 0.3.

### 2.8 Gut microbiota metagenomic analysis

Fecal samples from four randomly selected mice per group were subjected, and 50 mg of each fecal sample was used. All samples were collected using sterile plastic bags and immediately placed on dry ice for transport to the laboratory. They were stored at −80 °C for high-throughput DNA sequencing analysis.

Total genomic DNA was extracted from mouse fecal samples using the Mag-Bind^®^ DNA Kit (Omega Bio-tek, Norcross, GA, USA), with procedures strictly adhering to the manufacturer‘s instructions. The concentration and purity of the extracted DNA were determined using the TBS-380 kit (Omega Bio-tek) and the Drop2000 (Thermo Fisher Scientific) spectrophotometer, respectively. DNA extract quality was checked on 1% agarose gel.

DNA extract was fragmented to an average size of about 350 bp using Covaris M220 (Gene Company Limited, China) for paired-end library construction. Paired-end library was constructed using NEXTFLEX Rapid DNA-Seq (Bioo Scientific, Austin, TX, USA). Adapters containing the full complement of sequencing primer hybridization sites were ligated to the blunt end of fragments. Paired-end sequencing was performed on Illumina NovaSeq (Illumina Inc., San Diego, CA, USA) at Majorbio Bio-Pharm Technology Co., Ltd. (Shanghai, China) using NovaSeq 6000 S4 Reagent Kit v1.5 (300 cycles) according to the manufacturer's instructions (www.illumina.com).

The data were analyzed on the free online platform of Majorbio Cloud Platform (www.majorbio.com).

### 2.9 Statistical analysis

All data are presented as the mean ± S.E values. Data were evaluated by one-way analysis of variance using SPSS 27.0 and GraphPad Prism 8.02 software and by determining differences between the means using Duncan's multiple-range test. Values were considered statistically significant when *P* < 0.05.

## 3 Results

### 3.1 TTFV attenuated HFD-induced weight gain

**Figure 1:**
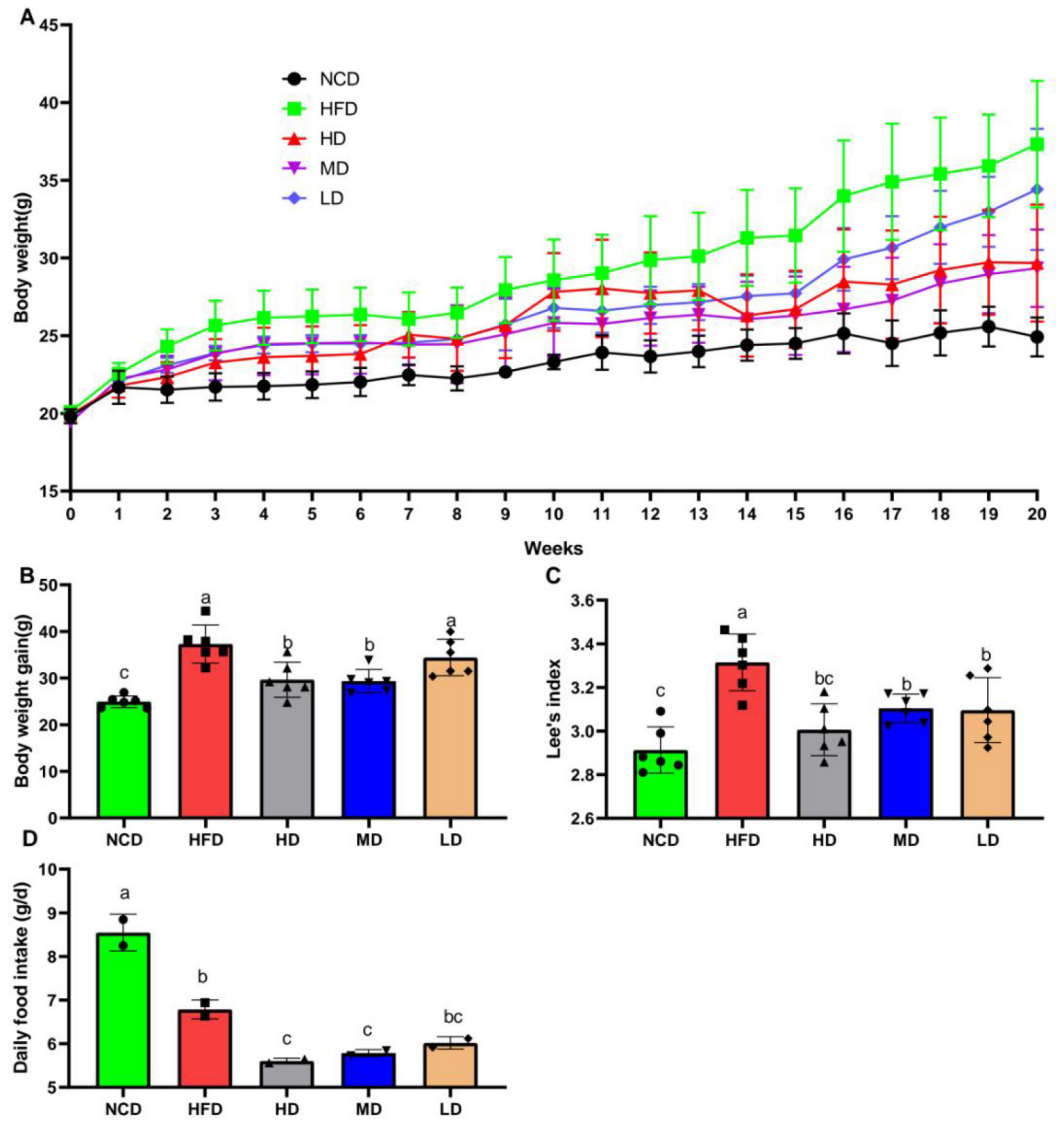
TTFV reduced body weight gain of HFD-fed mice (*n* = 6 per group). **(A)** Body weight changes during the experiment. **(B)** Final body weight. **(C)** Lee's index [calculated as body weight (g)^(1/3)^ × 1,000/body length (cm)]. **(D)** Daily food intake (*n* = 2 cages per group). Data are expressed as mean ± standard deviation (SD). Different letters (a–c) indicate significant differences based on one-way ANOVA and Duncan's *post hoc* test (*P* < 0.05).

As shown in Figure 1, the mean initial body weights of the five groups ranged from 20.78 to 23.67 g. One-way ANOVA revealed that baseline body weights did not differ significantly among the dietary groups (*F* = 1.348, *P* = 0.28). Body weight changes during the 20-week feeding period are shown in Figure 1A. Final body weights were significantly higher in the HFD group compared to the NCD group (*P* < 0.05). Supplementation with TTFV at high (HD) and medium (MD) doses resulted in significantly lower body weights compared to the HFD group (*P* < 0.05), while the low-dose (LD) group showed no significant difference (Figure 1B). Lee‘s index calculations revealed a similar pattern, with the HFD group exhibiting a significantly higher index compared to the NCD group (*P* < 0.05), which was dose-dependently reduced by TTFV administration (Figure 1C). Daily food intake measurements showed a dose-dependent decrease in food consumption in TTFV-supplemented groups compared to the HFD group (Figure 1D).

### 3.2 TTFV improved blood sugar levels

**Figure 2:**
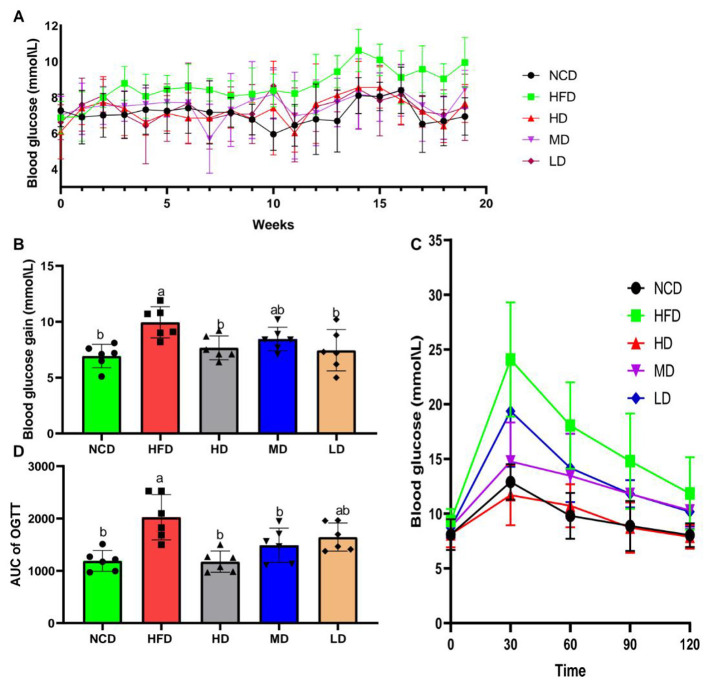
TTFV reduced blood glucose levels in HFD mice (*n* = 6 per group). **(A)** Weekly changes in blood glucose levels during the experiment. **(B)** Final blood glucose levels. **(C)** Fasting blood glucose levels. **(D)** Area under the oral glucose tolerance test (OGTT) curve (OCTT-AUC). Data are expressed as mean ± standard deviation (SD). Different letters (a–c) indicate significant differences based on one-way ANOVA and Duncan's *post hoc* test (*P* < 0.05).

To evaluate the glucose-lowering effect of TTFV, blood glucose levels in mice were monitored weekly during TTFV supplementation (Figure 2). Prolonged exposure to HFD led to a progressive increase in blood glucose levels. Levels began to rise after 3 weeks of HFD feeding and peaked at week 14. By week 19, the blood glucose levels in the HFD group were significantly higher compared to the NCD group (*P* < 0.05; Figures 2A, B). In contrast, TTFV supplementation counteracted this rise, with significant reductions observed only in the HD and LD groups, not the MD group did not exhibit a significant effect. The oral glucose tolerance test (OGTT) further demonstrated that fasting blood glucose and the area under the OGTT curve (OGTT-AUC) were significantly higher in the HFD group compared to the NCD group (*P* < 0.05; Figures 2C, D), showing that HFD feeding induced glucose intolerance. Conversely, mice receiving TTFV at 10 or 20 mg/g/d had significantly lower fasting blood glucose levels and OGTT-AUC compared to the HFD group (*P* < 0.05; Figures 2C, D). These findings demonstrate that TTFV can effectively mitigate HFD-induced blood glucose increase and improve glucose tolerance in mice.

### 3.3 TTFV improved blood lipid level

**Figure 3:**
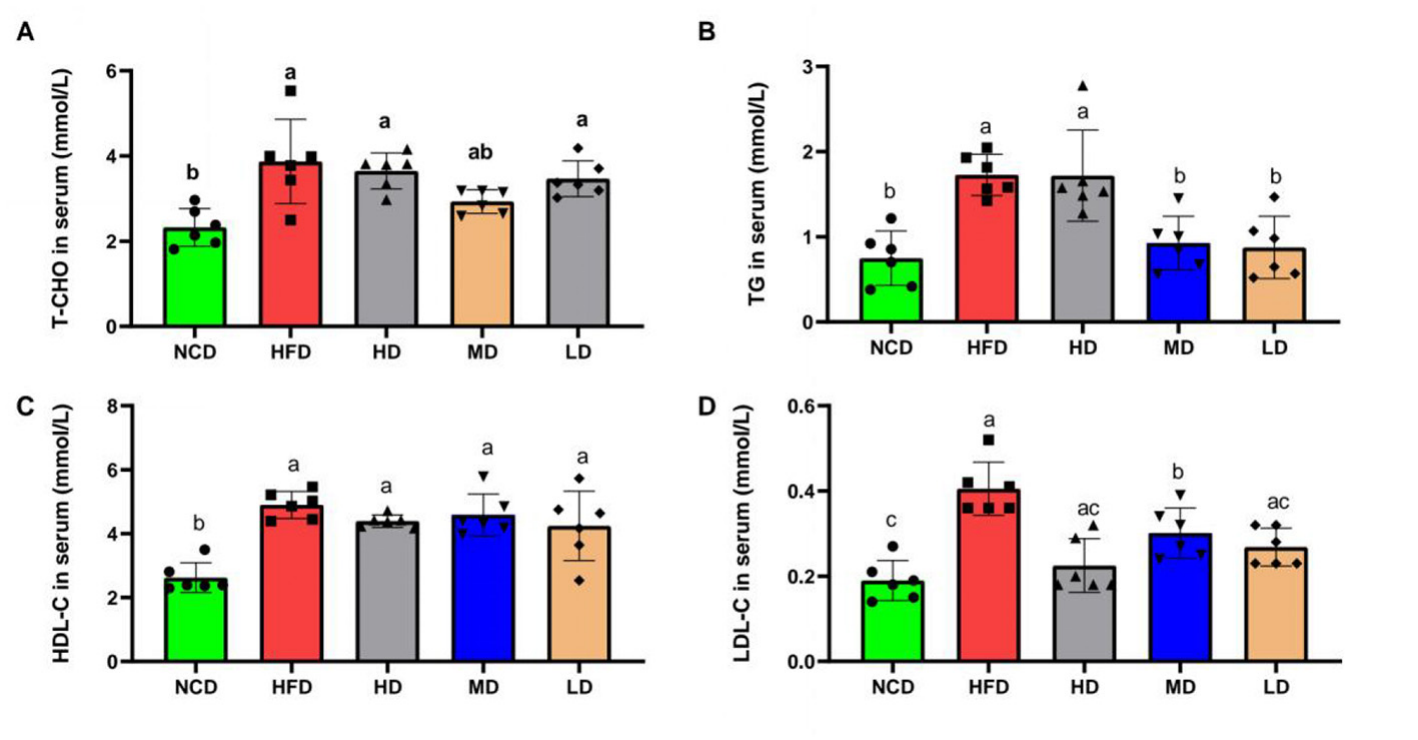
TTVF improved blood lipid levels in HFD-fed mice (*n* = 6 per group). **(A)** Total cholesterol (T-CHO). **(B)** Triglycerides (TG). **(C)** High-density lipoprotein cholesterol (HDL-C). **(D)** Low-density lipoprotein cholesterol (LDL-C). Data are expressed as mean ± standard deviation (SD). Different letters (a–c) indicate significant differences based on one-way ANOVA and Duncan's *post hoc* test (*P* < 0.05).

To assess the effect of TTFV on blood lipid levels, this study measured the serum concentrations of total cholesterol, triglycerides, and low-density lipoprotein cholesterol. Compared with the NCD group (*P* < 0.05), the HFD group showed significantly higher levels of T-CHO, TG, and LDL-C (Figures 3A–D). In HFD-fed mice, TTFV supplementation tended to reduce T-CHO levels, although the reduction in the MD group was not statistically significant (Figure 3A). TTFV significantly decreased TG and LDL-C levels (*P* < 0.05; Figures 3B, D). Additionally, HDL-C levels were reduced following TTFV supplementation (Figure 3C).

### 3.4 TTFV attenuated HFD-induced secretion of inflammatory factors

**Figure 4:**
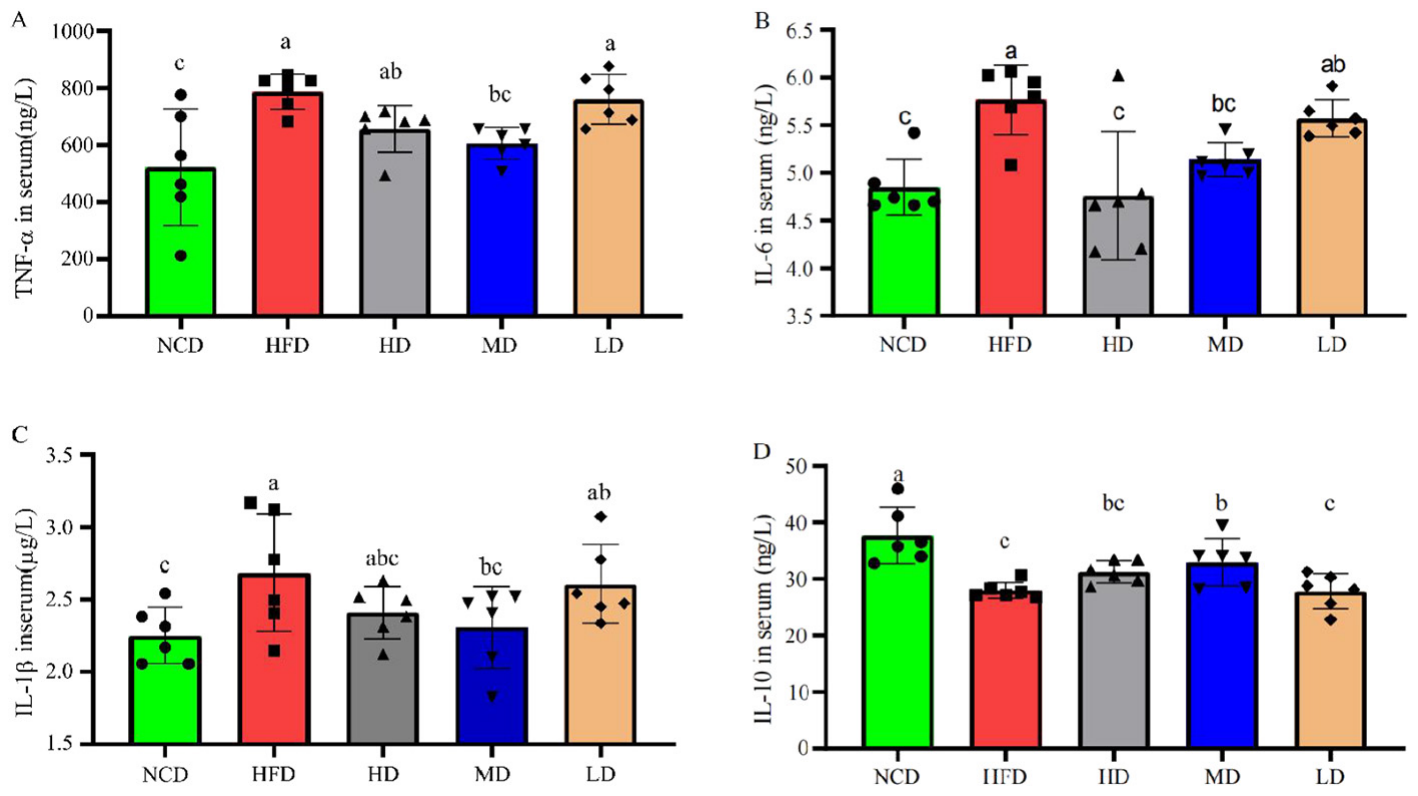
TTFV inhibited pro-inflammatory cytokine levels in HFD mice (*n* = 6 per group). Serum levels of **(A)** tumor necrosis factor alpha (TNF-α), **(B)** interleukin 6 (IL-6), **(C)** interleukin 1β (IL-1β), and **(D)** interleukin 10 (IL-10). Cytokine levels were measured by ELISA. Data are expressed as mean ± standard deviation (SD). Different letters (a–c) indicate significant differences based on one-way ANOVA and Duncan's *post hoc* test (*P* < 0.05).

As shown in Figure 4, compared with the control group, HFD feeding exacerbated inflammation by upregulating the expression of pro-inflammatory factors TNF-α, IL-6, and IL-1β (*P* < 0.05), while downregulating the expression of anti-inflammatory factor IL-10 (*P* < 0.05; Figures 4A–D). Notably, following TTFV supplementation, varying degrees of reduction were observed in the levels of pro-inflammatory factors: compared with the HFD group, medium-dose TTFV intervention significantly decreased the expression of TNF-α, IL-6, and IL-1β. Additionally, the expression level of IL-10 in the MD group was higher than that in the HFD group (*P* < 0.05; Figure 4D). These findings suggest that TTFV mitigates HFD-induced inflammation by upregulating the expression of IL-10 while suppressing the expression of TNF-α, IL-6, and IL-1β.

### 3.5 TTFV attenuated HFD-induced liver injury and hepatic steatosis

**Figure 5:**
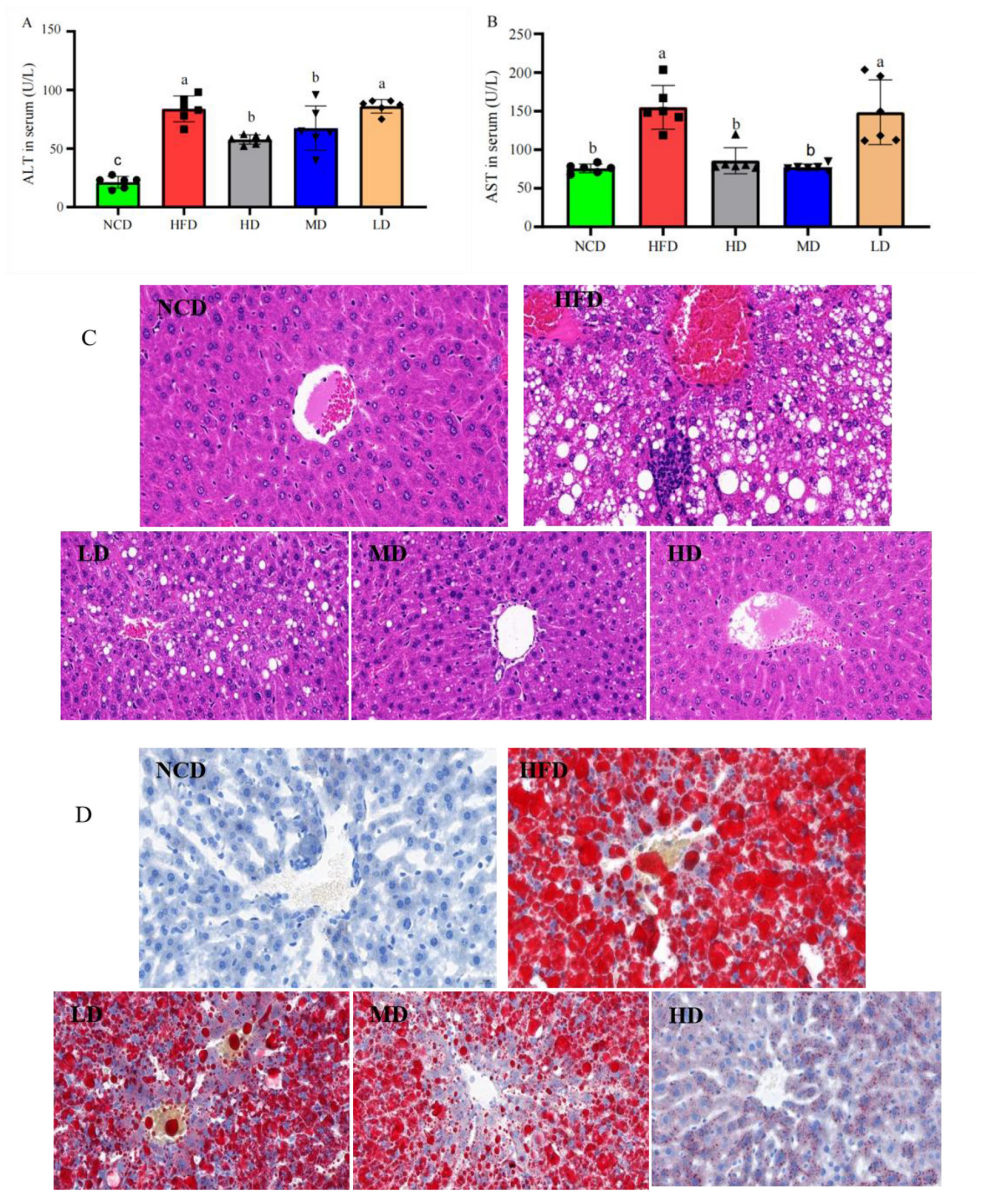
TTFV alleviated HFD-induced liver injury and hepatic steatosis (*n* = 6 per group). Serum levels of **(A)** alanine aminotransferase (ALT), **(B)** aspartate aminotransferase (AST). **(C)** H&E staining of liver section showing hepatic histopathology (scale bar = 200 μm). **(D)** Oil Red O staining of liver sections showing hepatic lipid deposition (scale bar = 200 μm). Data are expressed as mean ± standard deviation (SD). Different letters indicate significant differences based on one-way ANOVA and Duncan's *post hoc* test (*P* < 0.05).

To verify the hepatoprotective effect of TTFV against a high-fat diet (HFD), the serum indicators was analyzed of the mouse model. The results revealed that, compared to the NCD group, the serum levels of ALT and AST were significantly elevated *(P* < 0.05) in the HFD group (Figures 5A, B), indicating that a long-term HFD feeding induced hepatic injury and functional abnormalities. Additionally, supplementation with TTFV at medium and high doses (the HD and MD groups) significantly reduced the serum ALT and AST levels, which were elevated in the HFD group (*P* < 0.05; Figures 5A, B). These results suggest that administered at specific doses, it effectively alleviates HFD-induced liver injury. Histological analysis supported the above results. H&E staining (Figure 5C) revealed that liver cells in the NCD group exhibited normal morphology, with tightly arranged hepatocytes without fat vacuoles. These are indicators of normal liver metabolic function. Conversely, the liver cells in the HFD group had enlarged and irregular nuclei, loosely arranged hepatocytes with widened intercellular spaces, numerous visible fat vacuoles, and abundant fat droplets. These are indicators of abnormal liver metabolism. TTFV treatment improved these abnormalities in a dose-dependent manner. The MD and LD groups showed reduced disorganization within hepatocytes, narrower intercellular spaces, fewer fat vacuoles, and the absence of fat droplets. By contrast, the cell nuclei in the HD group were largely restored, with tighter radial cell organization and markedly reduced fat vacuoles and cellular damage. These findings further corroborate that HFD led to substantial structural and functional liver damage, and Yunnan large-leaved tea tree flower vinegar could effectively reverse the damage.Oil Red O staining further confirmed these effects (Figure 5D). HFD-fed mice exhibited severe hepatic steatosis, characterized by an increased number and size of fat droplets and disrupted cellular architecture. Compared to the HFD group, hepatic steatosis in the HD group was reduced, as indicated by the decreased number and size of fat droplets and the normal appearance of cellular structure. The reduction in hepatocyte steatosis in the LD group was comparable to that in the HD and MD groups. Quantitative analysis of Oil Red O staining showed that the positive area ratio was 75.13% in the NCD group, 0.46% in the HFD group, 64.45% in the LD group, 48.93% in the MD group, and 6.91% in the HD group (Table S3). These results strongly indicate that TTFV is capable of ameliorating HFD-induced liver tissue injury and hepatic steatosis.

### 3.6 TTFV attenuated HFD-induced oxidative stress

**Figure 6:**
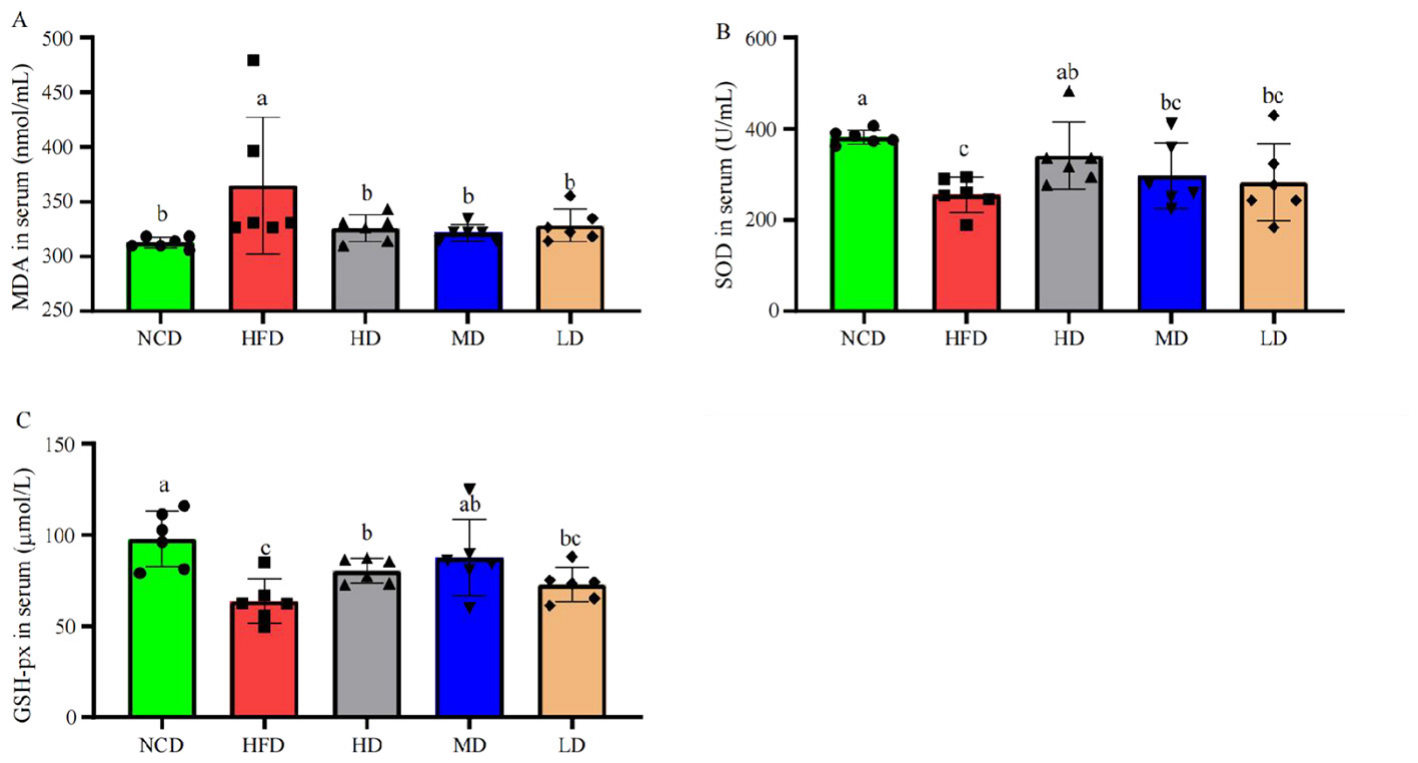
TTFV attenuated oxidative stress in HFD-fed mice (*n* = 6 per group). Serum levels of **(A)** malondialdehyde (MDA), **(B)** superoxide dismutase (SOD) activity, and **(C)** glutathione peroxidase (GSH-Px). Data are expressed as mean ± standard deviation (SD). Different letters (a–c) indicate significant differences based on one-way ANOVA and Duncan's *post hoc* test (*P* < 0.05).

To investigate the antioxidant effects of TTFV, this study measured relevant oxidative stress markers. As shown in Figure 6, MDA levels in the HFD group were significantly elevated (*P* < 0.05) compared to the NCD group, while both GSG-Px and SOD levels were significantly decreased (*P* < 0.05). These findings suggest that HFD feeding induced oxidative stress in mice. TTFV supplementation reversed these changes. Compared with the HFD group, MDA levels in the HD, MD and LD groups were reduced by 10.75, 11.89, and 10.00%, respectively (*P* < 0.05; Figure 6A). GSH-Px levels in the HD and MD groups increased by 11.54 and 20.09% (*P* < 0.05), respectively (Figure 6C), while the SOD activity in the HD group was enhanced by 25.16% (*P* < 0.05; Figure 6B). These results clearly indicate that TTFV promotes free radical scavenging and improve the oxidant defense capacity of HFD-fed mice.

### 3.7 Metabolomics analysis

**Figure 7:**
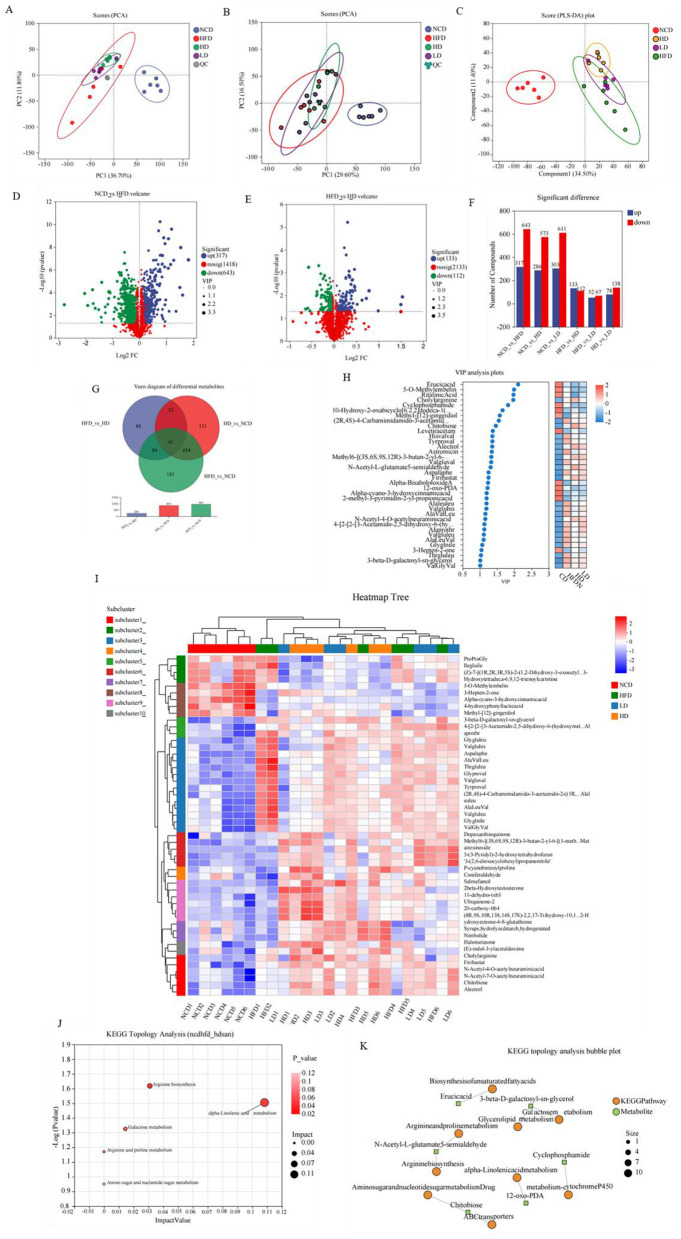
Effects of TTFV on metabolic profiles HFD-fed mice (*n* = 6 mice per group). **(A)** PCA score plot of QC samples, cationic mode. **(B)** PCA score plot of QC samples, anionic mode. **(C)** PLS-DA plot for multigroup comparisons. **(D)** Volcano plot, NCD vs. HFD. **(E)** Volcano plot, HFD vs. HD. **(F)** Expression difference statistics. **(G)** Venn diagram of differential metabolites. **(H)** VIP analysis plots. **(I)** Heatmap of metabolite clustering. **(J)** KEGG enrichment analysis network. **(K)** KEGG topology analysis bubble plot.

To evaluate the influence of TTFV on metabolic characteristics, metabolites in mouse feces were determined by liquid chromatography-mass spectrometry (LC-MS) in this study. The PCA score plots (Figures 7A, B) showed tight clustering of the QC samples, indicating good repeatability and reliable data quality. The NCD, HFD, HD, and LD groups showed clear separation trends, suggesting that HFD altered endogenous metabolic profiles and that TTFV gavage modified these profiles. Subsequent multigroup comparisons were performed, and PLS-DA plots revealed significant separation between the NCD and HFD groups, as well as distinct clustering among the HFD, HD, and LD groups (Figure 7C). Potential marker metabolites were identified using OPLS-DA with a threshold of VIP> 1 and *P* < 0.05, where the metabolites were considered to be significantly different. In total, 960 differential metabolites were identified between the NCD and HFD groups, while 246 were identified between the HD and HFD groups (Figures 7D–F). Venn analysis was conducted on these differential metabolites. The relative content changes in shared differential metabolites in the NCD, HFD, and HD groups were compared and analyzed using the mean content of differential metabolites in each group as a reference. The analysis revealed that 41 metabolites overlapped among the NCD, HFD, and HD groups and showed significant regression in the HD group after TTFV intervention (Figure 7G). Of these, 24 metabolites were significantly upregulated, and 17 were significantly downregulated. These differential metabolites showed clear intergroup differentiation and strong intragroup clustering, which suggests that TTFV intervention could modulate the metabolic profiles. A metabolic set was constructed using the 41 common metabolites, and the 30 most abundant metabolites were visualized in heatmaps. VIP analysis identified 36 metabolites with VIP> 1, consistent with the clustering results. In the HD group, metabolites, including Val-Gly-Val, 3-β-D-galactosyl-sn-glycerol, Val-Glu-Leu, and Asp-Ala-Phe, were downregulated (Figures 7H, I).

Pathway enrichment analysis was performed on the 41 differential metabolites (Figures 7N, J). A total of five metabolic pathways were identified: α-linolenic acid metabolism, arginine biosynthesis, galactose metabolism, arginine and proline metabolism, and amino sugar and nucleotide sugar metabolism (Figures 7H, I).

### 3.8 TTFV remodeled the gut microbiota of HFD-fed mice

**Figure 8:**
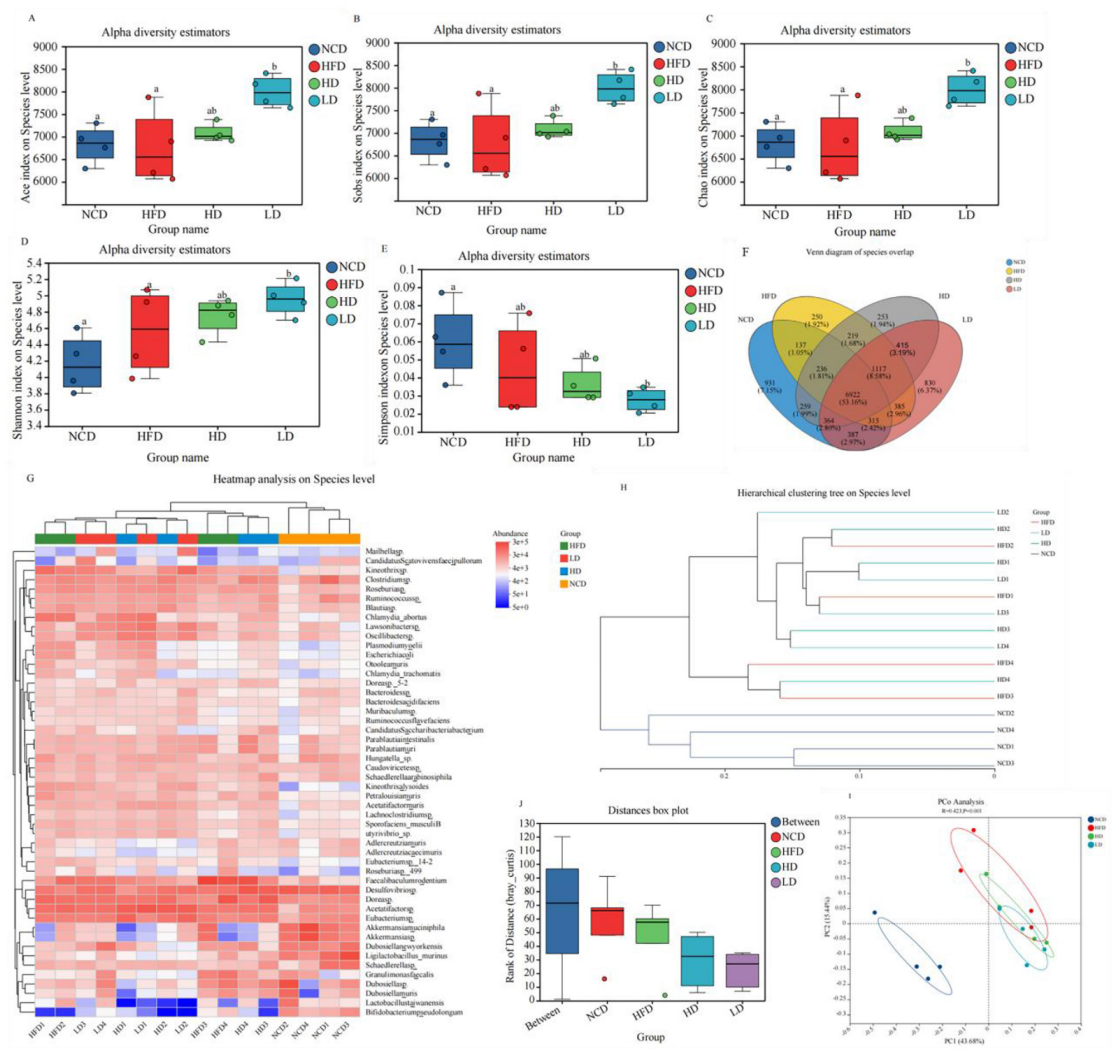
TTFV altered the gut microbiota diversity and composition in HFD-fed mice (*n* = 4 per group). **(A)** ACE index. **(B)** Sobs index. **(C)** Chao index. **(D)** Shannon index. **(E)** Simpson index. **(F)** Venn diagram of species overlap. **(G)** Heatmap showing the richness of the top 50 species. **(H)** Hierarchical clustering plot. **(I)** Principal coordinate analysis (PCoA). **(J)** ANOSIM similarity analysis. Data are expressed as mean ± standard deviation (SD). Different letters (a–c) indicate significant differences based on one-way ANOVA and Duncan's *post hoc* test (*P* < 0.05).

To evaluate the effect of TTFV on the intestinal microbiota of HFD-fed mice, a total of 1,830,065 sequences were obtained from 16 cecal samples by macrogenomic sequencing. Compared to the NCD group, richness indices of the gut microbiota (Sobs, Chao, and Ace) were decreased in the HFD group, although the decrease was not statistically significant (*P* > 0.05). Notably, the richness increased in the HD group relative to the HFD group (*P* > 0.05), and the LD group showed a significantly higher microbiota richness index (*P* < 0.05; Figures 8A–C). The Simpson index was lower in the HFD group compared to the NCD group, suggesting that HFD may disturb the gut microbiota composition. Moreover, Shannon indices in both the HD and LD groups were greater than in the HFD group, indicating that TTFV could enhance the diversity of species in the gut microbiota (Figures 8D, E). Venn diagrams revealed that 6,922 species were common to all four groups, with the NCD group having the largest number of unique species (931; Figure 8F). Heatmap analysis indicated that TTFV substantially altered the structural composition of the gut flora (Figure 8G). β-diversity analyses, including hierarchical clustering and principal coordinate analysis (PCoA), indicated that each of the four groups had a distinct flora structure. The HFD-associated groups were clustered more closely together (Figures 8H, I). Analysis of similarity (ANOSIM) further confirmed the significance of these clustering patterns (*R* = 0.422743, *P* = 0.001; Figure 8J).

**Figure 9:**
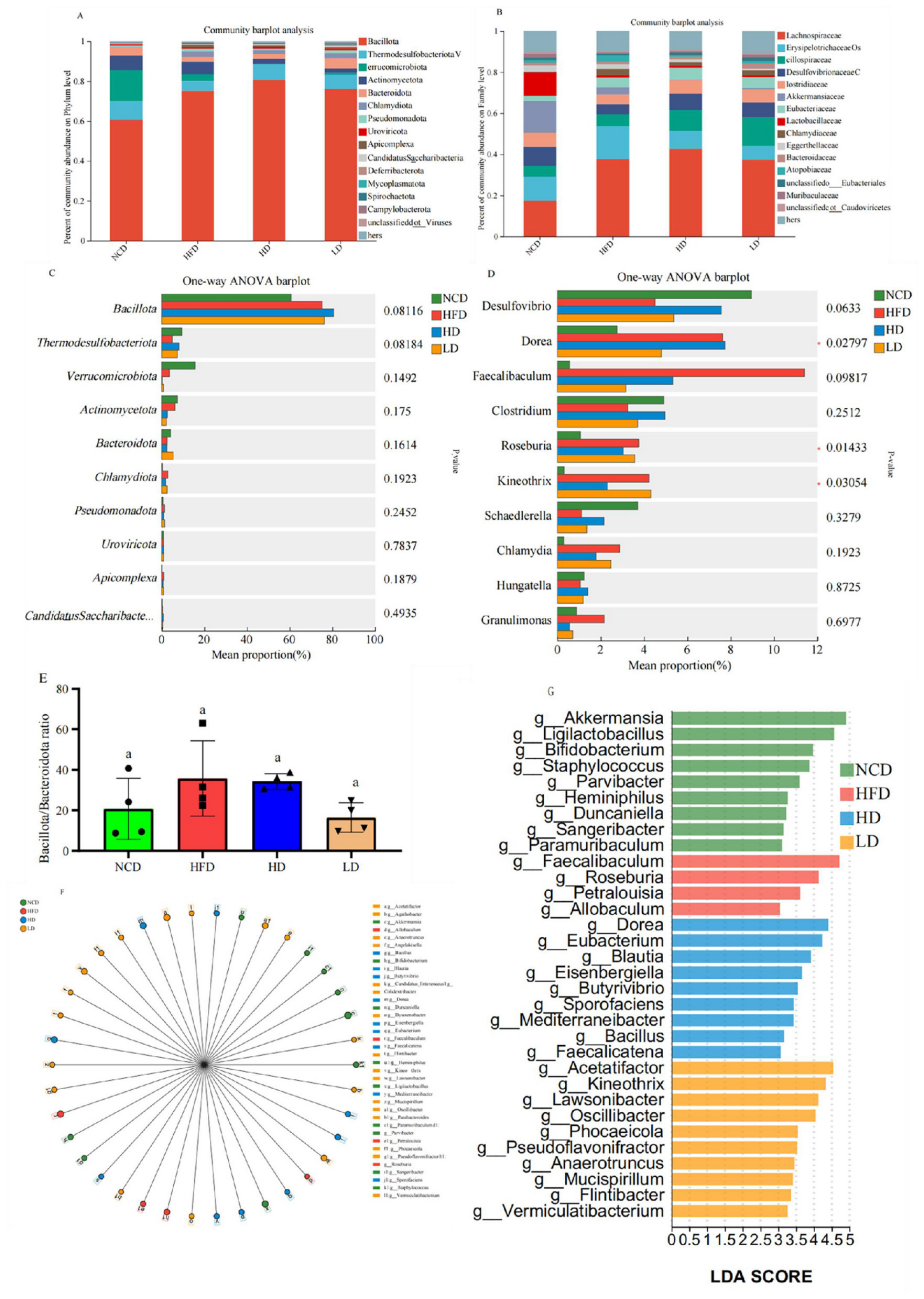
TTFV modulated the relative abundance of gut microbiota at the phylum, family, and genus levels in HFD-fed mice (*n* = 6 mice per group). **(A, B)** Relative abundance at the phylum **(A)** and family **(B)** levels. **(C, D)** Comparison of the relative abundance at the phylum **(C)** and genus **(D)** levels. **(E)** *Firmicutes*/*Bacteroidetes* ratios. **(F)** LEfSe analysis of different taxa. **(G)** Bar graph showing the LDA values (≥ 3.0) of significantly different taxa. Different letters (a–c) indicate significant differences based on one-way ANOVA and Duncan's *post hoc* test (*P* < 0.05).

At higher taxonomic levels, TTFV was found to have regulatory effects across the phylum, family, and genus levels. At the phylum level, HFD significantly increased the relative abundance of Bacillota (Figures 9A, C). In contrast, TTFV supplementation increased the richness of Thermodesulfobacteriota and Bacteroidota while decreasing the abundance of Chlamydiota, Pseudomonadota, and Apicomplexa (Figure 9C). Furthermore, our results showed that HFD elevated the *Bacillota*/*Bacteroidota* ratio, and TTFV decreased this ratio (Figure 9E), restoring microbial balance. At the family level, TTFV supplementation reduced the abundance of Erysipelotrichaceae while increasing the abundance of Oscillospiraceae, Desulfovibrionaceae, Clostridiaceae, and Eubacteriaceae (Figure 9B). At the genus level, TTFV increased the abundance of *Desulfovibrio, Clostridium, Schaedlerella*, and *Hungatella*, while decreasing the abundance of *Dorea, Faecalibaculum, Roseburia, Kineothrix, Chlamydia*, and *Granulimonas*.

LEfSe analysis further identified key taxa altered by TTFV supplementation. At a log LDA score threshold of 3, the abundance of 38 taxa was statistically significantly different (Figure 9F). HFD feeding affected 13 genera, with 4 genera enriched and 9 reduced, compared to the NCD group (Figure 9G). The most enriched genera in the HFD group included *Faecalibaculum, Roseburia, Petralouisia*, and *Allobaculum*. In contrast, TTFV intervention significantly modulated 23 genera, with 19 enriched and 4 reduced (Figure 9F). Among all, *Acetatifactor* and *Dorea* were enriched, while *Faecalibaculum* and *Roseburia* were suppressed.

### 3.9 Potential relationship between gut microbiota and related biomarkers

**Figure 10:**
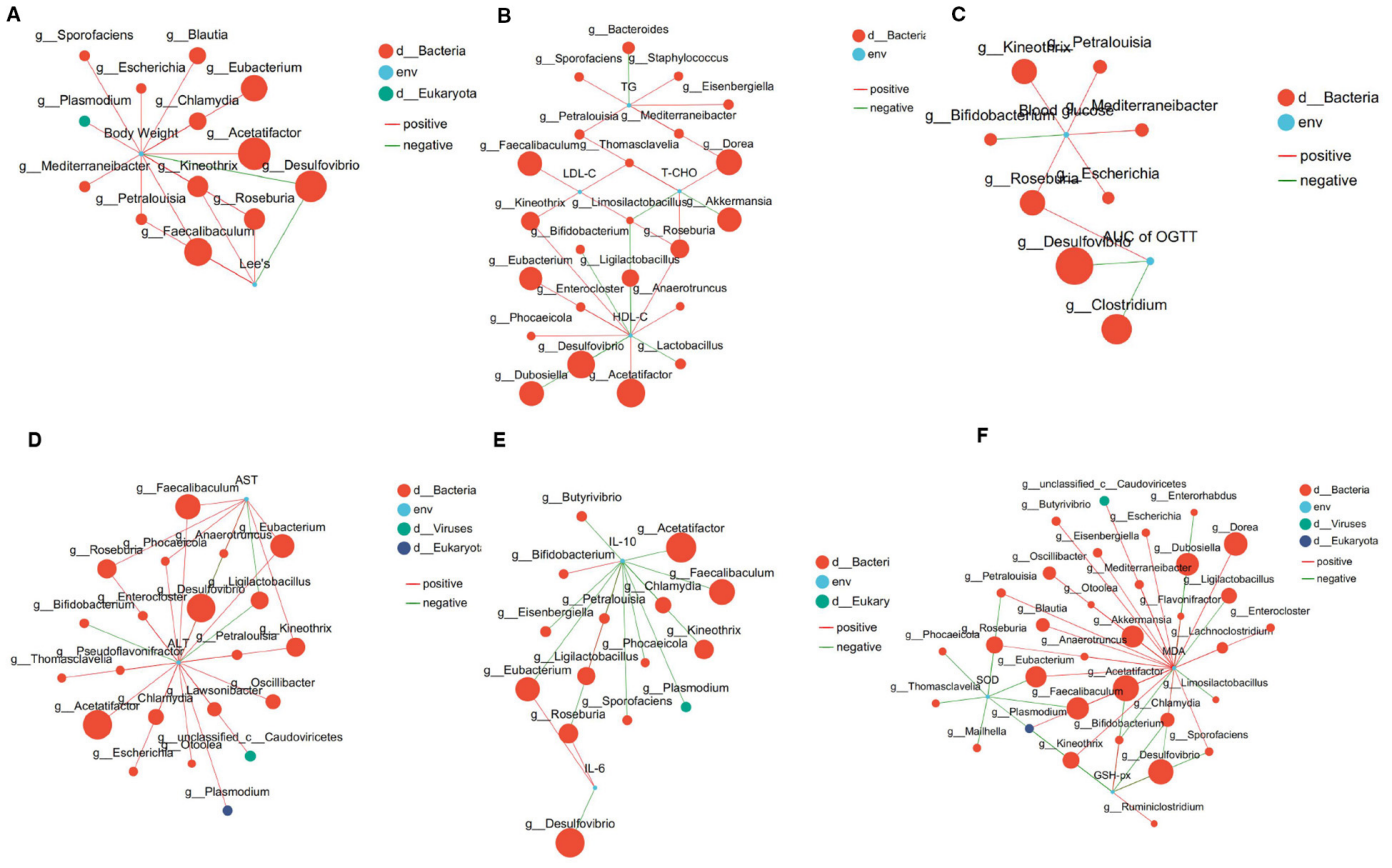
Correlation network analysis between gut microbes and biomarkers associated with metabolic syndrome (*n* = 6 per group). **(A)** Correlations with body weight and Lee's index. **(B)** Correlations with serum lipid profile (TG, T-CHO, LDL-C, and HDL-C). **(C)** Correlations with glucose homeostasis (fasting glucose and OGTT-AUC). **(D)** Correlations with liver injury indicators (ALT and AST). **(E)** Correlations with inflammatory factors (IL-10, IL-6, TNF-α, and IL-1β). **(F)** Correlations with oxidative stress indicators (MDA, SOD, and GSH-Px). Correlation strength was assessed by Spearman's coefficient (|*r*| ≥ 0.5, *P* < 0.05).Red lines represent positive correlations (*r* ≥ 0.5), and green lines represent negative correlations (*r* ≤ −0.5).

A two-factor correlation network analysis was conducted to examine the relationship between gut flora and obesity-related parameters. As shown in Figure 10A, 13 bacterial genera of gut bacteria were significantly correlated with body weight and Lee‘s index. *Desulfovibrio* was the only genus negatively correlated with the two parameters, whereas *Faecalibaculum, Roseburia, Petralouisia*, and *Kineothrix* showed significant positive correlation.

Figure 10B shows the correlation between intestinal bacterial genera and lipid synthesis-related parameters. Specifically, HDL-C exhibited significant correlations with 13 genera, whereas LDL-C was correlated with only 5 genera. *Roseburia* was the only genus positively correlated with all three lipid parameters (T-CHO, HDL-C, and LDL-C). *Kineothrix, Dorea, Petralouisia*, and *Thomasclavelia* were positively correlated with HDL-C and LDL-C. However, *Limosilactobacillus* was negatively correlated with T-CHO and HDL-C. Other genera, including *Bacteroides, Eisenbergiella, Akkermansia, Eubacterium*, and *Acetatifactor*, were associated with only a single lipid parameter.

Correlations with glucose homeostasis are shown in Figure 10C. Six genera, including *Petralouisia, Escherichia*, and *Roseburia*, were positively correlated with blood glucose. *Roseburia* was positively correlated with both blood glucose and OGTT-AUC. *Bifidobacterium* was negatively correlated with blood glucose, while *Clostridium* and *Desulfovibrio* were negatively correlated with OGTT-AUC.

Figure 10D shows the correlation between the gut microbiota and liver injury indicators. *Roseburia, Faecalibaculum, Eubacterium, Phocaeicola*, and *Kineothrix* showed positive correlations with ALT and AST, whereas *Ligilactobacillus* showed a significant negative correlation. Among the most abundant taxa, *Acetatifactor, Desulfovibrio*, and *Faecalibaculum* were positively correlated with ALT.

Correlations with inflammatory factors are depicted in Figure 10E. Among the four inflammatory factors assessed, only IL-10 and IL-6 exhibited correlations with gut bacterial genera. Specifically, *Ligilactobacillusand Bifidobacterium* showed positive associations with IL-10, whereas *Butyrivibrio, Acetatifactor, Faecalibaculum, Kineothrix*, and 11 additional genera displayed negative correlations with IL-10. Notably, *Eubacterium* and *Roseburia*, which were negatively correlated with IL-10, showed significant positive correlations with IL-6.

Correlations with oxidative stress response indices are illustrated in Figure 10F. *Kineothrix* was correlated with SOD, MDA, and GSH-Px. *Roseburia, Eubacterium, Faecalibaculum, Petralouisia, Kineothrix*, and *Plasmodium* were negatively correlated with SOD but positively correlated with MDA. *Ruminiclostridium* was positively correlated with GSH-Px. Collectively, these results suggest that gut microbiota are closely linked not only to host phenotype (body weight) but also to serum lipid-related parameters, glucose metabolism, inflammatory responses, liver injury indicators, and oxidative stress.

## 4 Discussion

Obesity and its associated metabolic diseases have become a major global health burden, with projections indicating continued growth in prevalence (17, 18). This study demonstrates that tea vinegar TTFV, derived from Yunnan large-leaved tea flowers, significantly alleviates HFD-induced obesity in mice through weight reduction and metabolic regulation. These findings align with broader research on tea-based interventions; for instance, green tea catechins and oolong tea polyphenols have similarly shown anti-obesity effects in rats (19, 20). Notably, TTFV exhibits distinct advantages: while conventional tea extracts typically require high doses (≥200 mg/kg) to achieve metabolic benefits, TTFV is administered at 100 mg/kg, indicating that the fermentation of tea flower significantly enhances its bioactivity.TTFV supplementation reverses high-fat diet-induced dyslipidemia (lowering total cholesterol, triglycerides, and LDL-cholesterol) and hyperglycemia while reducing hepatic steatosis and oxidative stress. The dose-dependent protective effects observed in body weight changes and Li‘s index measurements (Figures 1A–D) align with vinegar-specific metabolic effects reported by Urtasun et al. (21), their study demonstrated that apple cider vinegar improves insulin sensitivity by activating AMPK. Tea polyphenols and tea polysaccharides are two primary blood-sugar-lowering compounds in tea leaves (22), with fermented tea reducing post-prandial blood glucose levels, Li G et al. (23) found that tea products with varying degrees of fermentation contain distinct functional components capable of inhibiting digestive enzymes, enhancing cellular glucose uptake, suppressing gluconeogenesis-related enzymes, reducing advanced glycation end-product (AGE) formation, inhibiting dipeptidyl peptidase-4 (DPP-4) activity, modulating gut microbiota, and alleviating inflammation associated with oxidative stress. Tian et al. (24) demonstrated that yellow-leaf green tea (YLGT) accelerates energy metabolism by activating the AMPK/CPT-1α signaling pathway, inhibiting the FAS/ACC/SREBP-1c pathway, reducing fat synthesis, and suppressing obesity.

In both the model group and the high-fat diet group, significantly elevated levels of TNF-α and IL-6 were accompanied by a concurrent decrease in hepatic ATP content, indicating that prolonged high-fat feeding induces liver injury and functional abnormalities. Chronic metabolic inflammation may impair mitochondrial oxidative phosphorylation function. Elevated proinflammatory cytokines such as TNF-α activate signaling pathways including JNK and NF-κB, inducing excessive mitochondrial reactive oxygen species (ROS) production and triggering an energy crisis in hepatocytes (25). TTFV uniquely modulates dual markers: lipid peroxidation markers (MDA) and antioxidant enzymes (SOD, GSH-Px). The gut microbiota is crucial for human health. Within the gut microbiota, acetate, propionate, and butyrate are primarily produced by anaerobic fermentation of indigestible carbohydrates by gut microbes (26, 27). Studies indicate that specific dietary interventions (e.g., prebiotics or fermented foods) can modulate short-chain fatty acid production (including acetate), thereby improving gut health and overall metabolic status (28, 29). The TTVF may stem from synergistic interactions between its polyphenolic components (e.g., epigallocatechin gallate) and fermentative metabolites (e.g., acetate). This mechanism may enhance intestinal absorption and bioavailability, thereby reducing chronic inflammation, lipotoxicity, and mitochondrial dysfunction (30, 31). Chen et al. (32) reported that intestinal microbial degradation of tea tree flower polysaccharides enhances their inhibitory activities against α-amylase and α-glucosidase. Ling (33) demonstrated that *C. sinensis* can prevent HFD-induced obesity and hyperlipidemia by suppressing fat accumulation. Research indicates that *Camellia* flowers contain representative components similar to those in tea leaves, such as catechins, caffeine, and amino acids (34). However, existing studies have primarily focused on the physiological activity of direct extracts from Assam tea plant (J. W. Mast.) Kitam flowers (e.g., polysaccharides and flavonoids) or the raw material itself, with insufficient attention given to the lipid-lowering potential of their fermented products.This study first demonstrates that fermented tea flower vinegar TTFV significantly reduces the *Firmicutes*/*Bacteroidetes* ratio (Figure 9E), consistent with Tian et al.'s (35) findings that YLGT exerts its effects through the following mechanisms: correcting gut microbiota imbalance, enriching short-chain fatty acid (SCFA)-producing bacteria and increasing intestinal SCFA content, activating G protein-coupled receptors, inhibiting the TLR4/NF-κB signaling pathway, strengthening tight junctions, and repairing the damaged intestinal barrier. Building upon this, the present work further correlates changes in microbial community structure with specific fermentative metabolites, offering a novel mechanistic perspective in this field. Previous literature indicates that tea polyphenols can directly scavenge reactive oxygen species (ROS) and inhibit lipid peroxidation (36, 37), corroborating our findings that TTFV reduces pro-inflammatory factors (TNF-α, IL-6) while enhancing glutathione peroxidase and superoxide dismutase activity. In addition, the synergistic effect between antioxidant activity and microbial regulation may be the reason why TTFV exhibits superior performance compared to non-fermented tea products, as the fermentation process can enhance the bioavailability of polyphenolic substances (38, 39). Regrettably, constrained by the limited availability of residual liver tissue samples, this study could not assess nitric oxide (NO), a key marker of oxidative stress. Future investigations should incorporate this parameter to systematically elucidate TTFV‘s local antioxidant mechanisms.

Intestinal microbiota plays a pivotal role in metabolic regulation through energy harvest, SCFA production, and immune modulation (40, 41). Our metagenomic analysis revealed TTFV significantly reshapes the gut microbial community—enriching Bacteroidetes and Oscillospiraceae while suppressing Erysipelotrichaceae—with direct implications for SCFA production and intestinal barrier integrity (Figure 10). This microbial remodeling aligns with Tian et al.'s (42, 43) findings that tea polyphenols (TP) enrich SCFA-producing bacteria via FFAR2/3 signaling to enhance tight junction protein expression (ZO-1, occludin). However, TTFV exhibits superior microbial regulatory effects: it increased Akkermansia muciniphila abundance by 6.8-fold vs. HFD controls, whereas TP supplementation in comparable models showed only a 2.3-fold increase (44). This enhanced enrichment may stem from TTFV's unique fermentation-derived metabolites, which serve as prebiotic substrates for mucin-degrading bacteria. Critically, TTFV reduced the *Firmicutes*/*Bacteroidetes* (F/B) ratio (Figure 9E), a key metabolic health indicator where high ratios correlate with energy harvest efficiency and obesity (45, 46).

Collectively, TTFV exerts multi-faceted metabolic benefits through interconnected mechanisms, gut microbiota remodeling (reducing the Firmicutes/Bacteroidetes ratio), and regulating metabolic disturbances by modulating key pathways such as galactose metabolism, amino sugar and nucleotide sugar biosynthesis, and arginine biosynthesis. Although TTFV itself has shown a certain degree of hypolipidemic and anti-inflammatory effects, there are still some key research areas to be further explored. Specifically, in terms of verifying the mechanism of action *in vivo*, future research should rely on advanced technologies such as sterile mouse models or fecal microbiota transplantation (FMT) to more clearly reveal the direct impact of bioactive metabolites derived from TTFV on metabolic processes. In addition, in terms of structural and functional properties, although metabonomic analysis has successfully identified several key differential metabolites (such as 3-β-d-galactosyl-sn-glycerol and chitosan), their specific mechanisms of action in the pathogenesis of obesity are not fully understood. Therefore, it is of great significance to accurately confirm the biological activity and molecular targets of TTFV through targeted chemical synthesis combined with strict functional experimental verification.

## 5 Conclusion

This study deeply explains how TTFV can exhibit significant anti obesity effects by reshaping intestinal microbial community structure and regulating related metabolic pathways. Here, we demonstrated for the first time that TTFV, fermented using Kombucha, effectively prevented weight gain, hepatic fat accumulation, hyperlipidemia, and chronic inflammation in HFD-induced obese mice.

## Data Availability

The data presented in this study are publicly available. The data can be found here: https://www.ebi.ac.uk/, accession PRJEB108262.
