# Supplementary material for: Flower vinegar prepared from Yunnan large-leaved tea tree prevents high-fat diet-induced obesity in mice by regulating gut microbiota
Source: Front Nutr. 2026 Mar 18;13:1749951. doi: 10.3389/fnut.2026.1749951 (PMC13038928; doi:10.3389/fnut.2026.1749951)
Supplement: Supplementary file 1 [file Table_1.DOCX]

***Supporting Information for***

# Flower vinegar prepared from Yunnan large-leaved tea tree prevents high-fat diet-induced obesity in mice by regulating gut microbiota

Wenshu Peng^a,b^, Wei Yanga, Li Ma^a,b^, Qiaomei Wangc, Ruijuan Yangd, Aibin Ji^a,b^, MingyueShea, Tao Wanga, Wanying Gong^a,b^, Liang Yanb,*****

a*Puer Institute of Pu-Erh Tea, Pu’er 665000, Chin*a

b*College of Pu’er Tea, West Yunnan University of Applied Sciences, Pu’er 665000, China*

c*College of Tea Science, Yunnan Agricultural University, Kunming 650201, China*

d*College of Food Science and Technology, Yunnan Agricultural University, Kunming 650201, China*

*****Corresponding author.

E-mail addresses: 1190145@wyuas.edu.cn; jacky_4680@163.com

1. **Materials and methods**

1.1 In Vitro Assay for the Lipid-Lowering Inhibition Rate of TTFV

The processing method follows Tian Yu ^[1]^. Add 20 μL each of tea tree flower vinegar, vinegar, and apple cider vinegar, followed by 50 μL α-glucosidase solution (0.04 U/mL) and 280 μL phosphate buffer (67 mmol/L). Incubate in a water bath at 37°C for 5 minutes. Next, add 50 μL of PNPG solution (0.5 mmol/L). Gently mix to combine, maintain the water bath temperature at 37°C, and allow the reaction to proceed for 15 min. Then, add 200 μL of Na₂CO₃ (0.2 mol/L) to terminate the reaction. Allow to stand at room temperature for 5 minutes. Measure the absorbance at 405 nm and record as Sample A. Use distilled water as the blank control. Follow the same procedure for other reagents, measuring the absorbance of A blank and A background. The calculation formula is as follows:

$$\text{Inhibition Rate}\text{(\%)=}\frac{\text{A}_{\text{Blank}\text{-}}\left( \text{A}_{\text{Blank}}\text{-}\text{A}_{\text{Background}} \right)}{\text{A}_{\text{Blank}}}\text{×100\%}$$

1.2 In Vitro Assay for the Blood Glucose–Lowering Inhibition Rate of TTFV

Following the experimental method described by Qi Jing et al. ^[2]^, prepare a clean conical flask. Accurately pipette 8 mL of olive oil emulsion and 5 mL of 0.1 mol/L pH 7.2 PBS buffer into the flask. Then, accurately pipette 3 mL of tea tree flower vinegar (pre-adjusted to pH 7.2) into the flask. Place the flask on a magnetic stirrer and mix thoroughly. Next, accurately pipette 1 mL of 0.20 mg/mL lipase solution into the flask and mix thoroughly. Subsequently, the mixture was incubated at 37°C in a constant-temperature shaking incubator for 1.5 hours. The enzymatic reaction was then terminated by adding 15 mL of 95% ethanol. Add 2 drops of phenolphthalein indicator to the solution and titrate with 0.02 mol/L NaOH to determine the amount of fatty acids produced. Conduct three parallel experiments and calculate enzyme activity using the following formula for enzyme activity inhibition rate:

$$\text{X}\text{=}\frac{\left( \text{V}_{\text{1-}}\text{V}_{\text{2}} \right)\text{×}\text{C}_{\left( \text{NaOH} \right)}}{\text{t}\text{×}\left( \text{C}_{\text{Enzyme}}\text{×}\text{V}_{\text{Enzyme}} \right)}\text{×100\%}$$

In the equation: X represents the enzyme activity of the sample, μmol/(min·mg); V₁ denotes the volume of standard NaOH solution consumed during titration of the sample, mL; V₂ indicates the volume of standard NaOH solution consumed during titration of the blank, mL; C__(NaOH)_ is the concentration of the standard NaOH solution, mol/L; t represents the reaction time, min; C__(enzyme)_ is the mass concentration of lipase, mg/ mL; V__(enzyme)_ is the added volume of lipase, mL.

$$\text{Inhibition Rate(\%)=}\frac{\text{X}_{\text{1}}\text{-}\text{X}_{\text{2}}}{\text{X}_{\text{2}}}\text{×100\%}$$

In the formula: Inhibition rate refers to the percentage inhibition rate of pancreatic lipase activity; X_1_ denotes enzyme activity in the control group, μmol/(min·mg); X_2_ denotes enzyme activity in the sample group, μmol/(min·mg). Replace tea tree flower vinegar with either regular vinegar or apple cider vinegar, following the same method, to compare lipid-lowering effects.

**2.Results**

2.1 In Vitro Lipid-Lowering Inhibition Rate of TTFV

As shown in Table S1, TFV, vinegar, and apple cider vinegar all exhibit inhibitory effects on α-glucosidase. TTTFV demonstrated an α-glucosidase inhibition rate of 77.57%±1.55%; vinegar showed an inhibition rate of 50.44%±1.74%; while apple cider vinegar exhibited an inhibition rate of 59.69%±2.04%. In summary, TTFV demonstrated a 27.13% higher inhibition rate than vinegar and a 17.88% higher rate than apple cider vinegar. The in vitro hypoglycemic efficacy ranked as follows: TTFV > apple cider vinegar > vinegar, with significant differences observed.

2.2 In Vitro Blood Glucose–Lowering Inhibition Rate of TTFV

As shown in Table S2, Tea tree flower vinegar, regular vinegar, and apple cider vinegar all exhibited inhibitory effects on lipase. In parallel experiments, the lipase inhibition rate for tea tree flower vinegar was measured at 62.78%±2.33%; for regular vinegar, it was 39.09%±2.13%; while apple cider vinegar exhibited a lipase inhibition rate of 48.45%±1.70%. In summary, the in vitro lipid-lowering efficacy of tea tree flower vinegar significantly outperformed commercially available vinegar and apple cider vinegar. Tea tree flower vinegar demonstrated a lipase inhibition rate 23.69% higher than vinegar and 14.33% higher than apple cider vinegar, representing statistically significant differences.

Table S1: In Vitro Lipid-Lowering Inhibition Rate of TTFV

| Group | Group 1 | Group 2 | Group 3 | Average | Error Range |
| --- | --- | --- | --- | --- | --- |
| TTFV | 64.25% | 61.92% | 62.17% | 62.78% | ±2.33% |
| Regular Vinegar | 40.34% | 38.72% | 38.21% | 39.09% | ±2.13% |
| Apple Cider Vinegar | 48.79% | 49.13% | 47.43% | 48.45% | ±1.70% |

Table S2: In Vitro Blood Glucose-Lowering Inhibition Rate of TTFV

| Group | Group 1 | Group 2 | Group 3 | Average | Error Range |
| --- | --- | --- | --- | --- | --- |
| TTFV | 77.11% | 78.58% | 77.03% | 77.57% | ±1.55% |
| Regular Vinegar | 49.67% | 51.41% | 50.24% | 50.44% | ±1.74% |
| Apple Cider Vinegar | 58.97% | 59.10% | 61.01% | 59.69% | ±2.04% |

Table S3: The oil-red O staining quantification data

| **Group** | **Positive Area** | **Total Area** | **Positive Area Ratio** |
| --- | --- | --- | --- |
| NCD | 1,466,990 | 1,952,640 | 75.13% |
| HFD | 8,945 | 1,952,640 | 0.46% |
| LD | 1,258,466 | 1,952,640 | 64.45% |
| MD | 955,465 | 1,952,640 | 48.93% |
| HD | 135,025 | 1,952,640 | 6.91% |

**Reference**

[1] Y. Tian.( 2021) Effects of Different Processing Methods on the Inhibition of α-Glucosidase and α-Amylase by Okra [D]. Jiangnan University.

[2] J. Qi, Q. Z. Wang, J. Yang et al.( 2020) Study on the Effects of Water Extracts from Northern and Southern Hawthorn on Human Endogenous Pancreatic Lipase Activity [J]. Chinese Food and Nutrition, 26(10): 54-59.
